# Polysialic Acid Modulates the Binding of External Lactoferrin in Neutrophil Extracellular Traps

**DOI:** 10.3390/biology8020020

**Published:** 2019-03-28

**Authors:** Andrea Kühnle, Thomas Lütteke, Kim F. Bornhöfft, Sebastian P. Galuska

**Affiliations:** 1 Institute of Reproductive Biology, Leibniz Institute for Farm Animal Biology (FBN), Wilhelm-Stahl-Allee 2, 18196 Dummerstorf, Germany; kuehnle@fbn-dummerstorf.de (A.K.); bornhoefft@fbn-dummerstorf.de (K.F.B.); 2 Institute of Biochemistry, Faculty of Medicine, Justus-Liebig-University, Friedrichstr. 24, 35392 Giessen, Germany; 3 Institute of Veterinary Physiology and Biochemistry, Justus-Liebig-University Giessen, Frankfurter Str. 100, 35392 Giessen, Germany; thomas@luetteke-online.de

**Keywords:** polysialic acid, lactoferrin, neutrophil extracellular traps, innate immune system

## Abstract

Neutrophil extracellular traps (NETs) are formed by neutrophils during inflammation. Among other things, these DNA constructs consist of antimicrobial proteins such as lactoferrin and histones. With these properties, NETs capture and destroy invading microorganisms. The carbohydrate polysialic acid (polySia) interacts with both lactoferrin and histones. Previous experiments demonstrated that, in humans, lactoferrin inhibits the release of NET and that this effect is supported by polySia. In this study, we examined the interplay of lactoferrin and polySia in already-formed NETs from bovine neutrophils. The binding of polySia was considered to occur at the lactoferricin (LFcin)-containing domain of lactoferrin. The interaction with the peptide LFcin was studied in more detail using groups of defined polySia chain lengths, which suggested a chain-length-dependent interaction mechanism with LFcin. The LFcin domain of lactoferrin was found to interact with DNA. Therefore, the possibility that polySia influences the integration of lactoferrin into the DNA-structures of NETs was tested by isolating bovine neutrophils and inducing NETosis. Experiments with NET fibers saturated with lactoferrin demonstrated that polySia initiates the incorporation of external lactoferrin in already-loaded NETs. Thus, polySia may modulate the constituents of NET.

## 1. Introduction

The immune system is flexible and multifaceted. For instance, its most abundant leucocytes, neutrophil granulocytes, are able to use different mechanisms to combat pathogens [1,2,3]. In addition to phagocytosis and the release of antimicrobial components through degranulation, a beneficial suicide has been described that involves the release of decondensed DNA, which is associated with antimicrobial molecules [4]. The explosive release of the loaded DNA results in the vast spread of a three-dimensional (3D) DNA meshwork that surrounds the pathogens [4,5]. This molecular web is called a neutrophil extracellular trap (NET), as summarized by Brinkmann and Zychlinsky [6]. Since the DNA is equipped with antimicrobial components, such as histones, neutrophil elastase, lactoferrin, lysozyme, and catalase, the trapped pathogens can be efficiently killed [4,5]. One of the main advantages of NET is that these molecules remain at this hot spot; thus, high concentrations of such antimicrobial mediators can be reached and/or retained. These high concentrations, while advantageous, also carry risk. For instance, extracellular histones are toxic not only for bacteria but also for endogenous cells [7,8,9]. Therefore, an exaggerated NET formation has been suggested to be associated with numerous pathologies [8,10,11,12,13,14,15,16,17,18]. However, several open questions and controversial opinions remain about the formation of NETs and their roles in health and disease, as summarized by Boeltz et al. [19].

Histones are one of the major protein fractions in NETs [6]. Distinct endogenous biomolecules, such as the carbohydrate polysialic acid (polySia), are able to bind histones and suppress their cytotoxicity in a chain-length-dependent manner [7,20,21,22,23,24,25]. PolySia, a linear homopolymer, consists of α2,8-linked sialic acid residues, and its chains can reach a degree of polymerization (DP) above 90 [26,27,28,29]. In several body fluids, including blood, milk, and semen, polySia chains are present with the required DP for histone binding [20,30,31,32].

In these liquids, polySia is associated with a second important player of the innate immune system: lactoferrin [32]. Lactoferrin is a multifunctional immune protein continuously secreted by epithelial cells that also occurs in neutrophilic granules [33,34,35,36,37,38,39]. The diverse roles of lactoferrin are still being discussed; however, due to its high iron affinity, one of its most accepted antimicrobial characteristics is its ability to trap iron [34,35,40,41]. This creates iron-deficient areas and reduces bacterial growth. The same process may also occur in the case of NETs, since NETs are also loaded with lactoferrin by neutrophils during their formation. Lactoferrin was described to form a shell around activated neutrophils suppressing the release of NETs [42]. Thus, external lactoferrin might represent an inhibitory biomolecule for the formation of NETs [42]. We observed that polySia supports this effect of lactoferrin [32]. However, the interplay between lactoferrin and polySia in already-formed NET structures is unknown.

Since polySia binds not only lactoferrin but also histones of NETs, we hypothesize that it might be a potential modulator molecule of the accumulation of external lactoferrin in already existing NET fibers.

## 2. Materials and Methods

### 2.1. Materials

For all experiments, the reagents that were used were of analytical grade. To use polySia (colominic acid; GERBU, Heidelberg, Germany) in the cell culture experiments, lipopolysaccharides (LPS) were removed, as previously described [23].

### 2.2. Isolation of Bovine Neutrophils

The isolation of bovine neutrophils was slightly modified from Schuberth et al. [43]. Instead of a Ficoll solution, Histopaque-1077 (Sigma-Aldrich, Steinheim, Germany) was applied. RPMI (Roswell Park Memorial Institute) 1640 (Thermo Fisher Scientific, Waltham, MA, USA) supplemented with 1% penicillin/streptomycin (*v/v*) was used as the cell culture medium. Neutrophils were isolated from leftover blood samples of regulatory blood collections from other projects/for other medical reasons in addition to blood from slaughterhouse animals. The blood samplings, as well as the slaughter processes, were performed in accordance with applicable laws, relevant guidelines, and provisions for ethical regulations.

### 2.3. Polysialylated Fluorescent Bead Accumulation in NETs

Prior to the coupling reaction, fluorescent latex beads (amine-modified polystyrene, fluorescent red, Sigma-Aldrich) were washed twice, subsequently resuspended, and ultrasonically homogenized in 123 µL PBS (phosphate buffered saline). Next, 200 µg of dried polySia were dissolved in 25 µL PBS and added to the particles. We added 1.5 µL 5 M NaCNBH_3_, and the coupling reaction occurred for 2 h at 65 °C and 250 rpm. The received particles were washed twice and resuspended in 100 µL PBS.

The bovine neutrophils were seeded in a poly-L-lysine-coated 12-well chamber slide (22,500 cells per chamber). For the induction of NETosis, phorbol myristate acetate (PMA) (1.5 µM; Sigma-Aldrich) and calcium ionophore-ionomycin (3µM; Cell Signaling, Danvers, MS, USA) were added, and the cells were further incubated at 37 °C and 5% CO_2_ for 4 h. After being washed twice with PBS, to remove the remaining stimuli, the cells were incubated with 1.43 × 10^5^ polysialylated or unpolysialylated fluorescence particles/µL for 30 min at 37 °C in RPMI 1640. Afterward, the cells were fixed with 4% paraformaldehyde (PFA). Multiple further washes with PBS were conducted, and the cells were incubated with 0.5% Triton X-100 (Sigma-Aldrich) for 1 min at 20 °C, then washed again with PBS. Finally, nucleus staining was performed using 1 µg/mL 4′,6-diamidino-2-phenylindole (DAPI) (Carl Roth, Karlsruhe, Germany). All images were captured with a Zeiss Axio Imager A1 (Carl Zeiss, Oberkochen, Germany).

### 2.4. Native Gel Electrophoresis

For native gel electrophoresis, lactoferrin-biotin, and bovine (source: milk) as well as human (source: milk) lactoferrin (Sigma-Aldrich) were incubated with different concentrations of polySia as previously described [32,44]. PolySia was incubated either with bovine/human milk lactoferrin or bovine lactoferricin (LFcin) (B25; Bachem, Bubendorf, Switzerland) in 50 mM Tris buffer for 1 h at 30 °C with shaking. Lactoferrin was incubated with a mixture of different chain lengths (colominic acid), whereas LFcin was incubated with groups of defined chain lengths. To this end, polySia was fractionated by the DP, as described by Galuska et al. [22]. Native agarose gels (2%) were dissolved in 25 mM Tris/HCl (pH 8.5) running buffer containing 19.2 mM glycine [44]. Samples were separated for ~3.5 h (LFcin) or ~4.5 h (bovine or human lactoferrin) at 80 V. Proteins were fixed over night with 45% methanol:7.5% acetic acid (*v/v*) and were stained with Coomassie blue (Roti-Blue; Carl-Roth). 

### 2.5. Biotinylation of Lactoferrin

To synthesize the biotinylated lactoferrin, the bovine protein was dissolved in 2-N-morpholino ethanesulfonic acid (MES) buffer (100 mM; pH 4.7–5.5), and an equal volume of biotin hydrazide (50 mM; Sigma-Aldrich) was added. The protein’s carboxyl groups were activated through the addition of an 1-ethyl-3-(3-dimethylaminopropyl)carbodiimid (EDC) solution (Fluka; Sigma-Aldrich) to a final concentration of 2.5 mM (*w/v*, in the MES buffer). The biotinylation mixture was incubated for 2 h on a shaker. Afterward, the remaining EDC and unbound biotin hydrazide was removed through dialysis overnight in 20 mM NaHCO_3_ [45,46]. To test the biotinylation of the lactoferrin, the samples were denatured in Lane Marker Reducing Sample Buffer (Pierce, Thermo Fisher Scientific) for 5 min at 95 °C. The samples were run on a 13% sodium dodecyl sulfate polyacrylamide gel electrophoresis (SDS-PAGE) and either colored with Coomassie dye or blotted on a polyvinylidenfluorid (PVDF) membrane. Membrane-bound lactoferrin-biotin was detected using a horseradish peroxidase (HRP)-streptavidin conjugate (1:10,000; Rockland Immunochemicals, Inc., Limerick, PA, USA).

### 2.6. Enrichment of Lactoferrin-Biotin in Bovine NET Fibers

The bovine neutrophil cells were seeded in a poly-L-lysine-coated 12-well chamber slide (30,000 cells per chamber). NETosis was induced by adding 3 µM calcium ionophore-ionomycin and 150 nM PMA for 4 h at 37 °C in a CO_2_ incubator. The cells were fixated by adding 4% PFA (*w/v*) for 1 h. Afterward, three subsequent loading steps were performed for 1 h on a shaker at 37 °C, first with bovine lactoferrin (200 µg/mL), then with polySia (200 µg/mL), and finally with lactoferrin-biotin (200 µg/mL). The cells were permeabilized with 0.5% Triton X-100 (*v/v*) for 1 min and blocked with 2% IgG-free albumin (Carl-Roth). The lactoferrin-biotin was visualized with a murine monoclonal antibody against biotin that was fluorescein isothiocyanate (FITC)-conjugated (1:200; Jackson ImmunoResearch Inc., West Grove, PA, USA). The DNA was stained with DAPI (1 µg/mL). The NET cells were fixed again using 2% PFA (*w/v*) for 20 min. After these steps were completed, the cells were washed three times with PBS (pH 7.4). All images were taken with a Zeiss Axio Imager A1 (Carl Zeiss). The data from the three different experimental approaches were pooled. Each experiment was performed as a double determination, and three pictures per well were analyzed. Histograms of the FITC (green; lactoferrin-biotin)-colored pictures were analyzed to calculate the fluorescence intensity using ImageJ (Vision 1.51 s) (https://imagej.net/Contributors) [47]. 

### 2.7. Statistical Analyses

The data sets were analyzed using ANOVA, and Tukey’s tests were conducted for multiple comparisons using GraphPad Prism 7 software (GraphPad Software, San Diego, CA, USA). Statistical significance differences were set as not significant (n.s.), *p < 0.05, **p < 0.01, ***p < 0.001, and ****p < 0.0001.

### 2.8. 3D Simulations

For the 3D modeling of the lactoferrin-polySia interaction sites, YASARA software (YASARA Biosciences GmbH, Vienna, Austria) was used as previously described [23]. For human lactoferrin, the Protein Data Bank (PDB) entry code 1CB6 was used. PolySia chains with α2,8-linked Neu5Ac residues (DP 20) were designed previously [23]. In the molecular dynamics (MD) simulation, four polySia chains were placed at a distance of 20–25 Å to lactoferrin in a water box. The binding simulation, using YASARA and an AMBER03 force field [48], was calculated for 7 and 5.6 ns.

To compare the amino acid sequences of lactoferrin in humans, cattle, goats, camels, and horses, an alignment of the lactoferrin sequences was performed using the online tool Clustal Omega (www.ebi.ac.uk) with protein-sequences P02788, P24627, Q29477, Q9TUM0, and O77811 from UniProt database: (www.uniprot.org). To visualize the identities of the sequences, Jalview (2.10.5) (http://www.jalview.org/about/jalview-scientific-advisory-committee) was used [49]. In addition, a 3D structural alignment of human (PDB entry 1CB6) and bovine (PDB entry 1BLF) lactoferrin was calculated using YASARA.

## 3. Results and Discussion

### 3.1. Accumulation of PolySia Coupled to Red Fluorescent Particles in NET Fibers

**Figure 1:**
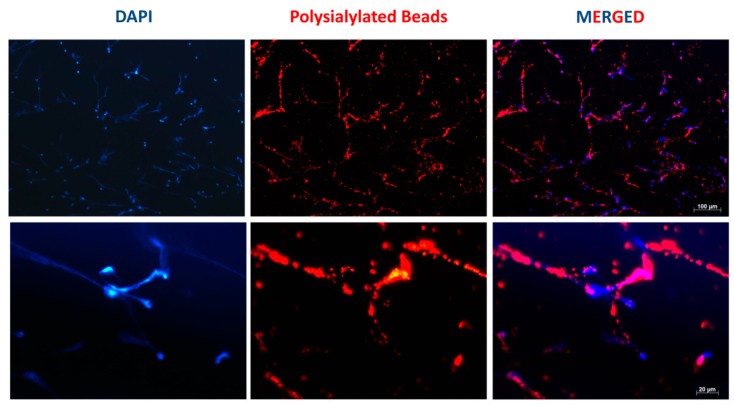
Polysialylated particles accumulating on the neutrophil extracellular trap (NET) fibers of bovine neutrophils. The neutrophils were isolated and stimulated with phorbol myristate acetate (PMA) and ionomycin to induce the formation of the NETs, and red fluorescent polysialylated particles were added. DNA staining was performed using 4′,6-diamidino-2-phenylindole (DAPI) (blue). Scale bars: 100 and 20 µm.

PolySia can mediate an accumulation of beads in NET, and this binding appears to be mediated by the interaction with histones [23]. However, in these experiments, the non-reducing ends of the chains were activated by oxidization, which led to a release of C8 and C9 of the carbon backbone in addition to the formation of an aldehyde group at C7. The resulting aldehyde group was used for the subsequent covalent coupling of the chemically modified polySia chains. In nature, however, a sialic acid polymer contains a free non-reducing end, since the other end—the reducing end—is attached to the glycoconjugates. This is also the case after the release of a polySia chain, for instance, due to a break in an internal linkage. In the first set of experiments, we investigated whether the naturally occurring form of polySia could also mediate the accumulation of beads into NET fibers, thus serving as an anchor molecule. To address this issue, polySia was coupled to red fluorescent latex particles using the reducing end. The resulting polysialylated beads were added to the NET filaments, which were produced by bovine neutrophils following stimulation with PMA and ionomycin. The DNA backbone of the NETs was visualized using DAPI. As shown in Figure 1, the polysialylated particles accumulated on the chromatin structures of the NETs. Unpolysialylated beads showed no specific accumulation on NET (Figure S1). Thus, independent of both its ends (reducing and non-reducing), a polySia chain can mediate the binding of its carrier to the NET. However, in the case of polysialylated proteins, the binding of polySia with its interaction partners may be modulated by several factors, such as the number of polySia chains per glycosylation site, the number of polysialylated glycosylation sites per protein backbone, and the localization at the protein backbone, in addition to the orientation of the polysialylated glycans.

### 3.2. Interaction of PolySia with LFcin Depends on Its DP

**Figure 2:**
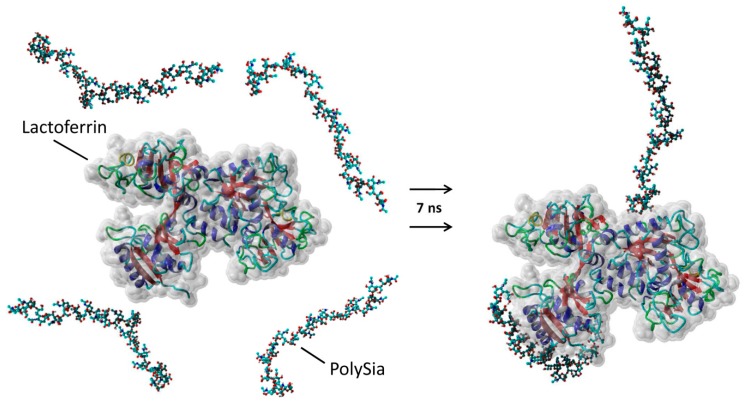
A molecular dynamics simulation of polysialic acid (polySia) binding to lactoferrin. Lactoferrin was placed with four polySia chains (20 sialic acid units) in a water box. After 7 calculated nanoseconds, the simulation showed two possible binding sites for polySia on the lactoferrin. Lactoferrin is displayed in a ribbon form with a dynamic surface. The polySia is shown in a ball-and-stick form, with hydrogen (gray), oxygen (red), carbon (turquoise), and nitrogen (blue) atoms.

Besides histones, lactoferrin is also present in NETs and may represent another binding partner for polySia. The interaction seems to occur at the LFcin-containing domain of lactoferrin, which is also a known DNA binding site [32,50,51,52]. To simulate a binding of polySia to lactoferrin, an MD simulation was calculated with the PDB structure of human lactoferrin and previously designed 20-degree long polySia chains [23]. In this simulation, four polySia chains were set in proximity to lactoferrin in a water box. After a calculated time of 7 ns, two of the chains were found to be in close contact with the lactoferrin (Figure 2 and Figure S2). The other two chains showed no migration to the protein backbone. One polySia chain interacted with the LFcin-containing domain of lactoferrin in the simulation (Figure S3). These results are in line with previous experiments showing that an antibody against LFcin inhibited the binding of polySia [32]. Regarding the MD simulation and the interaction area of the LFcin domain, the MD simulation is just a simulation and can only suggest a possible interacting mechanism. When we repeated the simulation, the polySia chains, which interact with the LFcin domain, were found in a different orientation (Figure S2b). Both models may only show the possibilities of initiating the interaction and not the final protein-carbohydrate complex. More detailed experimental analyses concerning the binding process (for example, using mutagenesis and co-crystallization) are necessary to determine the involved amino acid residues of LFcin in detail and to produce an unambiguous 3D model of the lactoferrin-polySia complex.

In addition to the LFcin domain, the first MD simulation suggested that a second binding site might be present (Figure S2a). However, in the second simulation, only the LFcin domain was targeted by polySia chains (Figure S2b). No additional experimental evidence for this second binding domain currently exists to proof the simulation. Thus, in contrast with the LFcin domain, no significant evidence exists to support the presence of a binding area for α2,8-linked sialic acid residues at this site. 

**Figure 3:**
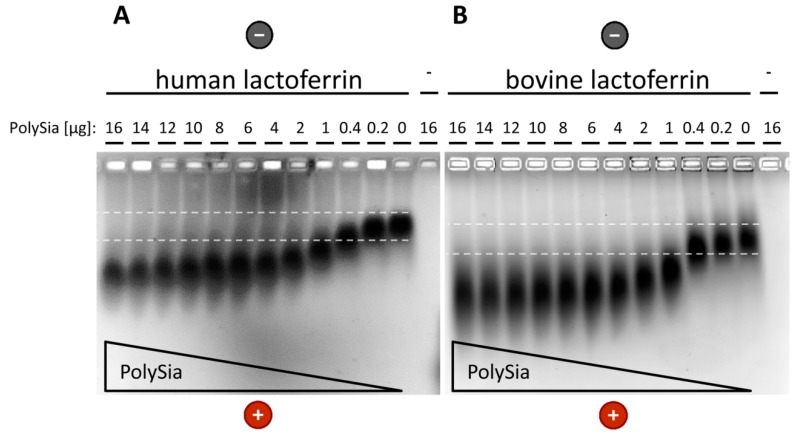
PolySia interacting comparably with (**a**) human and (**b**) bovine lactoferrin. The interaction of polySia with human and bovine lactoferrin was analyzed using a native agarose gel system. The lactoferrin (8 µg; molecular weight of lactoferrin: bovine, 87 kDa; human, 82 kDa) was incubated with different amounts of polySia and subsequently separated through electrophoresis. Proteins were stained using Coomassie blue.

The LFcin-containing domain is conserved in the lactoferrin of humans and farm animals that are commonly kept for milk production (Figures S4 and S5). Human milk, and especially bovine milk, are frequently used to purify lactoferrin for clinical products, functional food, and cosmetic products [53,54]; therefore, we chose to compare the interaction of polySia with human and bovine lactoferrin. To this end, human and bovine lactoferrin were separated using native gel electrophoresis in the presence and absence of various polySia concentrations. As shown in Figure 3, the lactoferrin of both species shows comparable migration characteristics in the presence of polySia. The changes in migration might reflect an altered total charge after the formation of protein/polySia complexes. 

**Figure 4:**
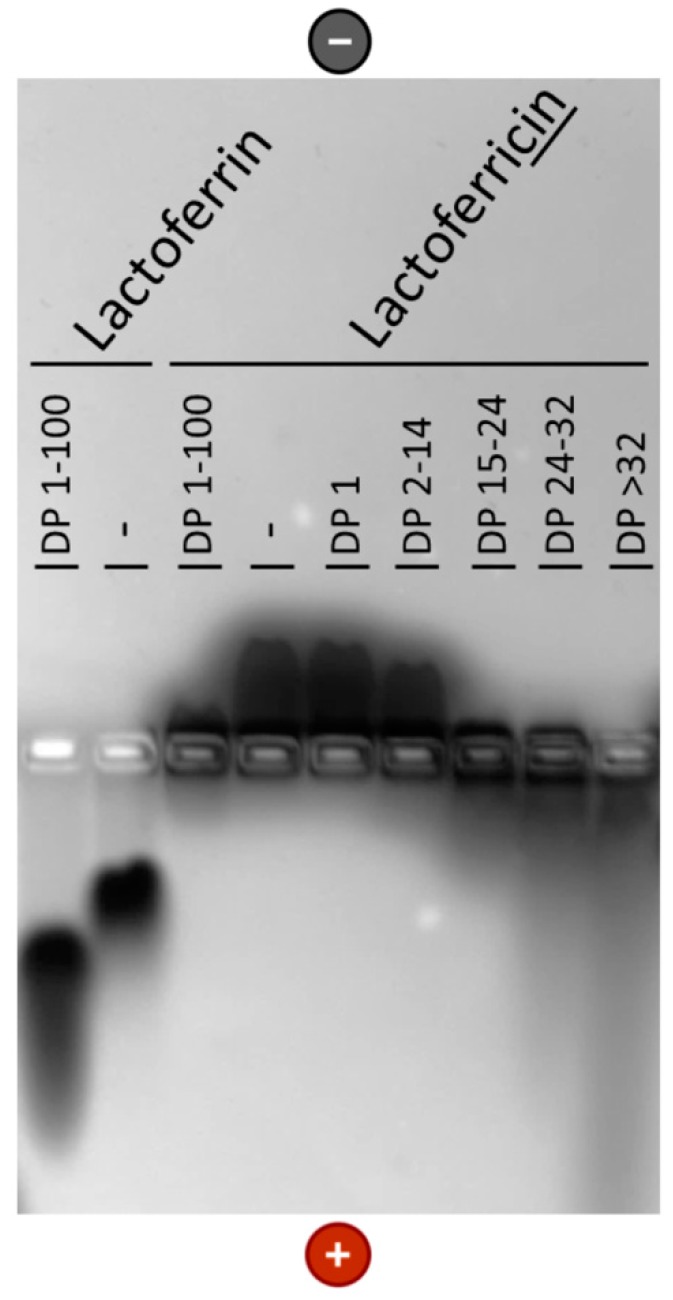
Lactoferricin (LFcin) interacts with polySia in a chain-length-dependent manner. Bovine LFcin (10 µg; molecular weight of 3 kDa) was incubated with polySia (5 µg) using groups with different degree of polymerization (DP). Subsequently, the samples were separated using native agarose gel electrophoresis. As positive control, lactoferrin was used (10µg). Proteins were stained with Coomassie blue.

We then studied the interaction with LFcin (representing the antimicrobial peptide fragment of lactoferrin) in more detail using polySia of different chain lengths. As shown in Figure 4, changes in the DP up to 14 had only a slight influence on the migration of bovine LFcin. In contrast, longer polySia chains significantly enhanced migration to the anode. This observation is in line with previously published experiments using lactoferrin [32] and supports the suggestion that polySia interacts with the LFcin-containing domain in a chain-length-dependent manner.

### 3.3. PolySia Supports the Enrichment of External Lactoferrin into NET Fibers

As previously mentioned, lactoferrin is an important component of NETs. The binding of DNA is mediated by the LFcin domain [50,51,52]. The same domain also interacts with polySia [32]. This suggests that the polySia and DNA of NETs may compete for the binding of lactoferrin, and long polySia chains, which already bind histones, may mediate the binding of external lactoferrin, as shown in polysialylated particles (Figure 1) [23]. 

To test whether polySia influences the integration of bovine lactoferrin into NETs, in vitro experiments were performed using isolated bovine neutrophils. Since the lactoferrin of neutrophils is already incorporated into NETs during NETosis, we wanted to use biotinylated lactoferrin to distinguish between the lactoferrin of neutrophils and the added lactoferrin fraction. 

**Figure 5:**
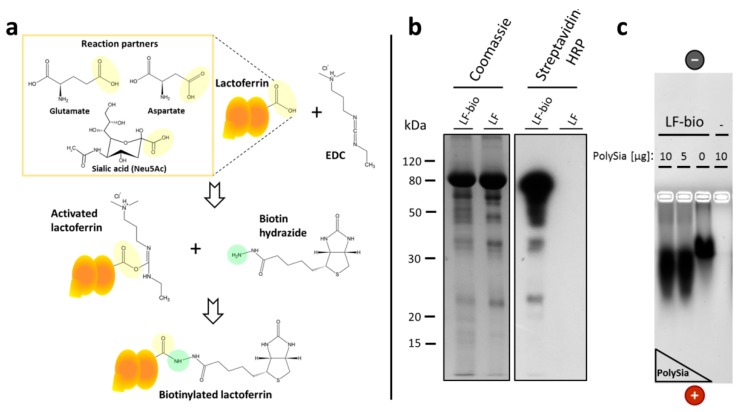
Biotinylation of bovine lactoferrin. (**a**) Bovine lactoferrin was biotinylated using 1-ethyl-3-(3-dimethylaminopropyl)carbodiimid (EDC) and biotin-hydrazide under acidic conditions. (**b**) Biotinylation of lactoferrin was controlled using Coomassie staining and streptavidin-horseradish peroxidase (HRP). Therefore, unbiotinylated lactoferrin (LF) and biotinylated lactoferrin (LF-bio) were used. (**c**) The biotinylated lactoferrin was incubated with different amounts of polySia and subsequently separated using native gel electrophoresis.

In the first set of experiments, the applicability of biotinylated lactoferrin was tested. The applied biotinylation of lactoferrin targets the acidic groups (Figure 5a). A linkage strategy was selected because prevalent basic amino acids may initiate the binding between lactoferrin and polySia as well as DNA. Biotin hydrazide can be linked to the carboxyl groups of proteins following activation with EDC (Figure 5a). Lactoferrin has three possible reaction partners: the amino acids glutamate and aspartate and the sialic acid residues of glycans. In the reaction, the carboxyl groups of the protein can form an unstable O-acylisourea-intermediate with the EDC’s carbodiimide reaction group. Through the addition of the primary amine biotin-hydrazide, the EDC is replaced by stable amide binding. 

At first, a successful biotinylation of lactoferrin was checked using SDS-gel electrophoresis (Figure 5b). Lactoferrin was visualized with Coomassie blue. The unmodified and biotinylated forms of the lactoferrin showed slightly different migration characteristics. The biotinylated form ran marginally higher, which may have been a result of the attached covalent biotin molecules.

The biotinylation was additionally tested through Western blotting using HRP-conjugated streptavidin (Figure 5b). As expected, the biotinylated lactoferrin showed a strong signal in contrast with the unbiotinylated lactoferrin, demonstrating that the lactoferrin was successfully biotinylated.

In a further experiment, the interaction between biotinylated lactoferrin and polySia was tested using native gel electrophoresis (Figure 5c). Here, biotinylated lactoferrin was incubated with different concentrations of polySia. The results indicated that polySia changed the migration of the biotinylated lactoferrin. Thus, complex formation is not influenced by biotinylation.

**Figure 6:**
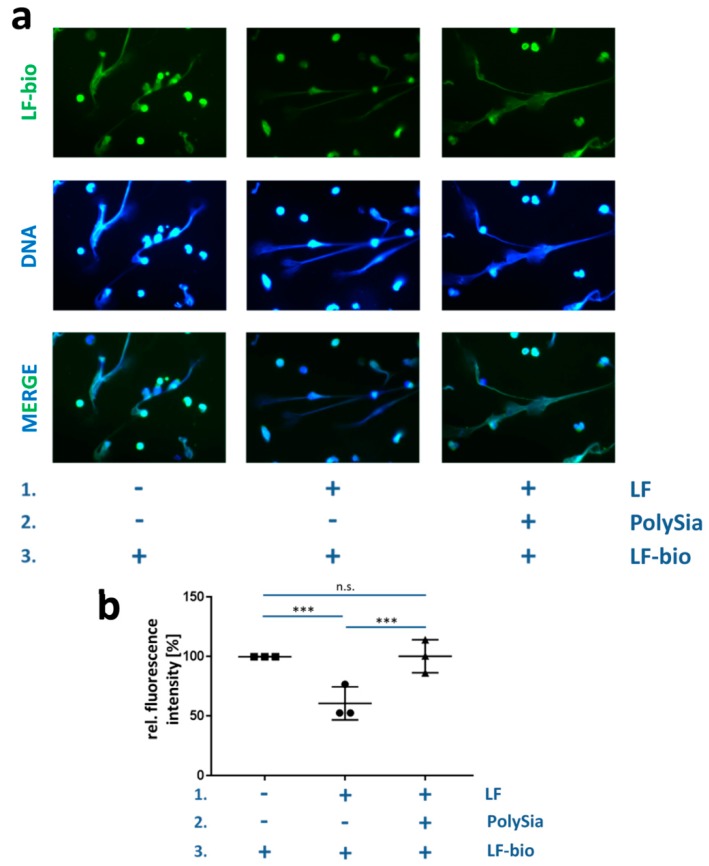
PolySia modulates the integration of lactoferrin into NETs. (**a**) After the formation of the NET fibers by induced bovine neutrophils, the fibers were treated in three successive steps: (1) Unbiotinylated lactoferrin, (2) polySia, and (3) lactoferrin-biotin. Three different combinations were applied. DAPI (blue) was used to stain the DNA, and lactoferrin-biotin (green) was visualized with an antibody against biotin. The scale bar represents 100 µm. (**b**) Quantification of lactoferrin-staining. The values for lactoferrin-biotin alone were set to 100%. Note: n.s. denotes not significant, *p < 0.05, **p < 0.01, and ***p < 0.001.

To address the question as to whether polySia can modulate the integration of external lactoferrin into exposed NET fibers, a binding assay with the biotinylated form was designed. In this assay, the NET fibers of stimulated bovine neutrophils were incubated with biotinylated lactoferrin. The binding of the biotinylated lactoferrin was visualized using FITC-conjugated antibodies against biotin. As expected, the biotinylated lactoferrin accumulated on the DNA fibers of the NETs (Figure 6a). The integration of biotinylated lactoferrin can be inhibited when its binding sites in NET are already blocked by lactoferrin before the application of biotinylated lactoferrin, resulting in a decreased fluorescence staining (Figure 6). Thus, in already loaded DNA, the integration of additional external lactoferrin is repressed.

However, the addition of polySia after the addition of native lactoferrin, but prior to the application of biotinylated lactoferrin, initiated the integration of novel lactoferrin into NETs. Due to the application of polySia, comparable staining intensities were determined in the case of unsaturated and presaturated NETs. This means that, overall, comparable amounts of biotinylated lactoferrin were assimilated (Figure 6b). Therefore, the previously presaturated NET was again able to uptake biotinylated lactoferrin, suggesting that polySia can create binding areas for external lactoferrin in NETs that have already been loaded with lactoferrin. Since the LFcin domain can bind both polySia and DNA, these results suggest that the DNA of NETs directly competes with polySia for lactoferrin binding. Thus, polySia and DNA are competitors for the same binding domain of lactoferrin and might dynamically modulate an exchange of lactoferrin by altering the interactions with the LFcin domain. 

However, as shown in Figure 1 for polysialylated beads, polySia chains can also act as molecular anchors for NETs when these chains are covalently coupled to a carrier. Therefore, another possibility might be that free long polySia chains can bind to more than one interaction partner, as mentioned above. Consequently, the binding of polySia to histones could create additional interaction possibilities for lactoferrin binding when enough free sialic acid residues of histone-bound polySia chains are still unoccupied. This would generate additional binding sites for lactoferrin apart from the DNA. However, no evidence exists for this type of mechanism at the moment.

## 4. Conclusions

One of the most important antimicrobial functions of lactoferrin is its ability to capture iron and make it unavailable to bacteria [34,35,40,41]. A dynamic and fluid interplay between lactoferrin, soluble polySia, polySia/histone-complexes, and DNA would allow an exchange of lactoferrin and thus a modulation of the constituents of NET. Via a switch to different binding partners, maturated lactoferrin might be replaced by external lactoferrin. Through this process, iron-deficient areas could be maintained to inhibit bacterial growth in NETs.

## Supplementary Materials

The following are available online at https://zenodo.org/record/2613411#.XJyW_KIRWUk: Figure S1: Unpolysialylated particles show no specific accumulation on the NET fibers of bovine neutrophils; Figure S2: Rotatable three-dimensional (3D) models of two independently simulated interactions between polySia and lactoferrin; Figure S3: Detailed depiction of the LFcin-containing domain of lactoferrin and the interacting polySia chain (MD simulation result); Figure S4: 3D structural alignment of human and bovine lactoferrin; and Figure S5: Sequence alignment of lactoferrin using the sequences of humans, bovine animals, goats, horses, and camels.
